# Comparison of accuracy between augmented reality/mixed reality techniques and conventional techniques for epidural anesthesia using a practice phantom model kit

**DOI:** 10.1186/s12871-023-02133-w

**Published:** 2023-05-20

**Authors:** Tatsuya Hayasaka, Kazuharu Kawano, Yu Onodera, Hiroto Suzuki, Masaki Nakane, Masafumi Kanoto, Kaneyuki Kawamae

**Affiliations:** 1 Department of Anesthesiology, Yamagata University Hospital, 2-2-2 Iidanishi, Yamagata City, Yamagata, 990-9585 Japan; 2 Department of Medicine, Yamagata University School of Medicine, Yamagata, Japan; 3 Critical Care Center, Yamagata University Hospital, Yamagata, Japan; 4 Department of Emergency and Critical Care Medicine, Yamagata University Hospital, Yamagata, Japan; 5 Department of Radiology, Division of Diagnostic Radiology, Yamagata University Hospital, Yamagata, Japan

**Keywords:** Epidural anesthesia, Augmented Reality/Mixed reality, Epidural Space puncture point, Image training, Technique Learning Tool

## Abstract

**Background:**

This study used an epidural anesthesia practice kit (model) to evaluate the accuracy of epidural anesthesia using standard techniques (blind) and augmented/mixed reality technology and whether visualization using augmented/mixed reality technology would facilitate epidural anesthesia.

**Methods:**

This study was conducted at the Yamagata University Hospital (Yamagata, Japan) between February and June 2022. Thirty medical students with no experience in epidural anesthesia were randomly divided into augmented reality (-), augmented reality (+), and semi-augmented reality groups, with 10 students in each group. Epidural anesthesia was performed using the paramedian approach with an epidural anesthesia practice kit. The augmented reality (-) group performed epidural anesthesia without HoloLens2Ⓡ and the augmented reality (+) group with HoloLens2Ⓡ. The semi-augmented reality group performed epidural anesthesia without HoloLens2Ⓡ after 30 s of image construction of the spine using HoloLens2Ⓡ. The epidural space puncture point distance between the ideal insertion needle and participant’s insertion needle was compared.

**Results:**

Four medical students in the augmented reality (-), zero in the augmented reality (+), and one in the semi-augmented reality groups failed to insert the needle into the epidural space. The epidural space puncture point distance for the augmented reality (-), augmented reality (+), and semi-augmented reality groups were 8.7 (5.7–14.3) mm, 3.5 (1.8–8.0) mm (P = 0.017), and 4.9 (3.2–5.9) mm (P = 0.027), respectively; a significant difference was observed between the two groups.

**Conclusions:**

Augmented/mixed reality technology has the potential to contribute significantly to the improvement of epidural anesthesia techniques.

**Supplementary Information:**

The online version contains supplementary material available at 10.1186/s12871-023-02133-w.

## Background

Epidural anesthesia, the gold standard for postoperative analgesia, is used with general anesthesia in thoracic, abdominal, pelvic, and lower extremity surgery. The analgesic effect of epidural anesthesia is superior to that of paravertebral nerve block, and it may be effective in reducing postoperative cognitive dysfunction and stress response [1–3]. However, epidural anesthesia is difficult. Clinicians must locate the epidural space by feeling the epidural needle tip with their finger and “walking” it, which is performed blindly and requires experience, tactile acuity, and anatomical knowledge.

The incidence of insertion failure and epidural anesthesia difficulty is approximately 7% and as high as 26% among anesthesia residents [4, 5]. These occurrences are attributed to obesity, atypical spine anatomy, or supraspinous ligament ossification due to advanced age [6–8]. Difficulty inserting an epidural catheter is increasingly painful for the patient. Multiple epidural punctures also cause local pain. The risk of nerve damage increases and the procedure may be interrupted if the patients are unable to maintain posture during the puncture [5, 9]. Additionally, epidural anesthesia is associated with complications such as intravascular cannula insertion (0.67%), unintentional dural puncture (0.61%), and epidural hematoma [10–12]. Blind “walking” procedures performed by inexperienced clinicians have a high potential for complications. However, there are few educational tools for learning this technique in epidural anesthesia, and training is difficult. Methods for safe and accurate epidural anesthesia have been explored extensively, including devices for epidural needles and ultrasound echo-based epidural and fiber optic-based techniques; however, few devices support epidural anesthesia [13–20].

Recent developments in augmented reality/mixed reality (AR/MR) technology have facilitated the visualization of the invisible and have been utilized in various fields. In medicine, they are used as educational tools for ventricular drainage and extracorporeal membrane oxygenation cannulation [21–24]. Specifically, head-mounted AR/MR devices, such as Hololens2Ⓡ, can project images constructed by computed tomography (CT) of the patient’s anatomy onto their body, which would aid in clinical settings and medical training [25].

The blind technique is the gold standard for epidural anesthesia. Since the practice process is often performed blind, it is important to understand the anatomy of the patient’s spine. We hypothesized that the use of AR/MR technology to visualize internal structures using spine images constructed from the patient’s own CT images and projected onto the patient’s back would help facilitate a smooth and accurate epidural anesthesia procedure. Since the current epidural anesthesia practice method is only a blinded method using an epidural anesthesia practice kit, the blinded technique (standard technique) was used as a control group, and this study aimed to examine the usefulness of using a tool (AR/MR technology) for visualization during the procedure. In addition, the purpose of this study was to examine the usefulness of using AR/MR technology as a tool for constructing images to improve the accuracy of the procedure. This study examined the possibility of using AR/MR for epidural needle placement in epidural anesthesia in accordance with textbooks and whether AR/MR technology can be used as a training tool for medical students (MSs) in epidural anesthesia techniques.

If the feasibility of epidural anesthesia using AR/MR is proven, difficulties during epidural catheter insertion can be avoided, and patient pain and anesthesia induction time can be reduced. Improved epidural anesthesia techniques may resolve issues with epidural anesthesia in obese patients and patients with atypical spinal anatomy or supraspinous ligament ossification.

## Methods

This study was approved by the Ethics Committee of Yamagata University and conducted in accordance with the Declaration of Helsinki (The approval numbers are 2021 − 346 and UMIN000046701). Written informed consent was obtained from all participants.

### Model

**Fig. 1:**
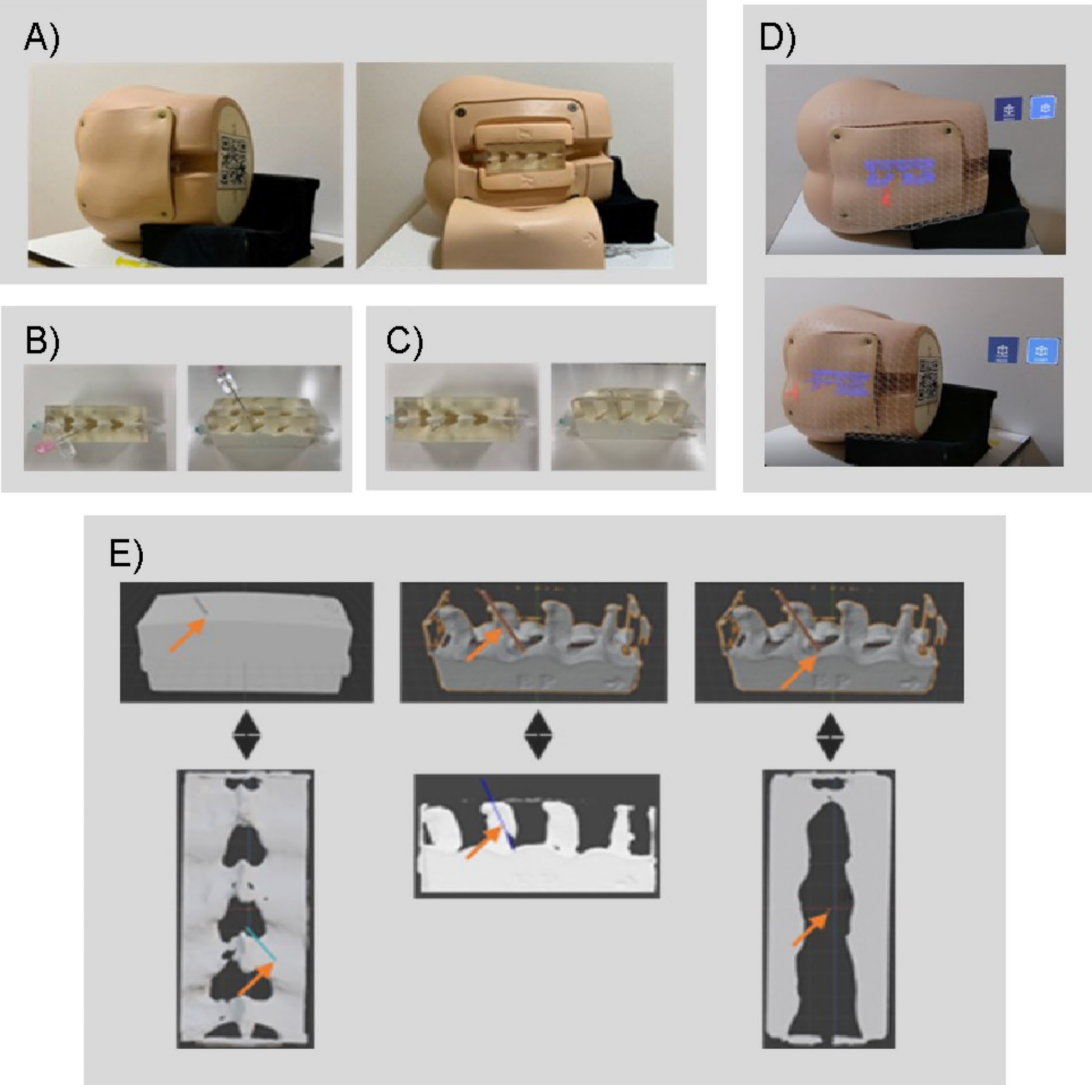
Epidural Anesthesia Practice Kit and ideal Insertion Model and Hologram Visibility. Remove the pad inside the epidural anesthesia practice kit (**a**). The epidural needle is inserted at the ideal point (**b**), and the needle is cut with nippers to create the ideal insertion model (**c**). An AR marker is attached to the head side of the epidural anesthesia practice kit. HoloLens2Ⓡis set to recognize the AR marker, and a hologram of the ideal insertion model is projected at the same position as the pad in the epidural anesthesia practice kit. The red holographic needle shows the ideal epidural needle insertion path (**d**). The view of the performer using HoloLens2Ⓡ is shown. The 3D model of the surface puncture point, puncture angle, and epidural space puncture point constructed from CT images and cross-sectioned in Blender 2.83.9 (**e**). Yellow arrows indicate the surface puncture point, puncture angle, and epidural puncture point

We used a commercially available phantom epidural anesthesia practice kit for the lumbar spine (Lumbar Puncture Simulator II; KYOTO Kagaku, Kyoto, Japan) equipped with silicone skin and air-filled epidural space, and an 18G Tuohy needle (PericanⓇ, B. Braun, Melsungen, Germany) (Fig. 1a). A CT scanner (Aquilion-One Vision, Canon Medical Systems Corporation, Otawara, Japan) created 0.5-mm cross-sectional images at 0.25-mm intervals for the construction of images of the epidural anesthesia practice kit. The images were processed for metal removal with metal artifact reduction algorithms to improve image quality and prevent obstructions in the area of interest. An ideal insertion model was captured when a needle was inserted into the epidural puncture pad inside the epidural anesthesia practice kit (Supplementary Fig. 1). The insertion method was determined to be in accordance with the textbook, where the needle entry point was 1 cm lateral and caudal to the space between the cephalic and caudal spinous processes, and the needle entry angle was 15° to the vertical; the needle was then “walked” cephalad and placed in the epidural space using the loss of resistance method (Fig. 1b) [26]. The epidural needle was cut with nippers (Fig. 1c). The hologram data sets of the ideal insertion model and epidural puncture pad were imported into the HoloLens2Ⓡ device (Microsoft Corp., Redmond, WA, USA) and stored as an application for use in epidural anesthesia. The AR marker was attached to the head side of the epidural anesthesia practice kit. HoloLens2Ⓡ was set to recognize the AR marker, and a hologram of the ideal insertion model was projected at the same location as the pad inside the epidural anesthesia practice kit (Fig. 1d). Vuforia SDK (PTC Inc., Boston, MA, USA) was used for the AR markers and Unity 2019.4.9 (Unity Technologies, San Francisco, CA, USA) for the holograms. A developmental toolkit for MR applications (MRTK 2.4.0, Microsoft Corp.) was also used. The operator was able to observe a hologram of the ideal insertion model projected on the back of the epidural anesthesia practice kit while using HoloLens2Ⓡ (Supplementary Fig. 2).

### Procedure

**Fig. 2:**
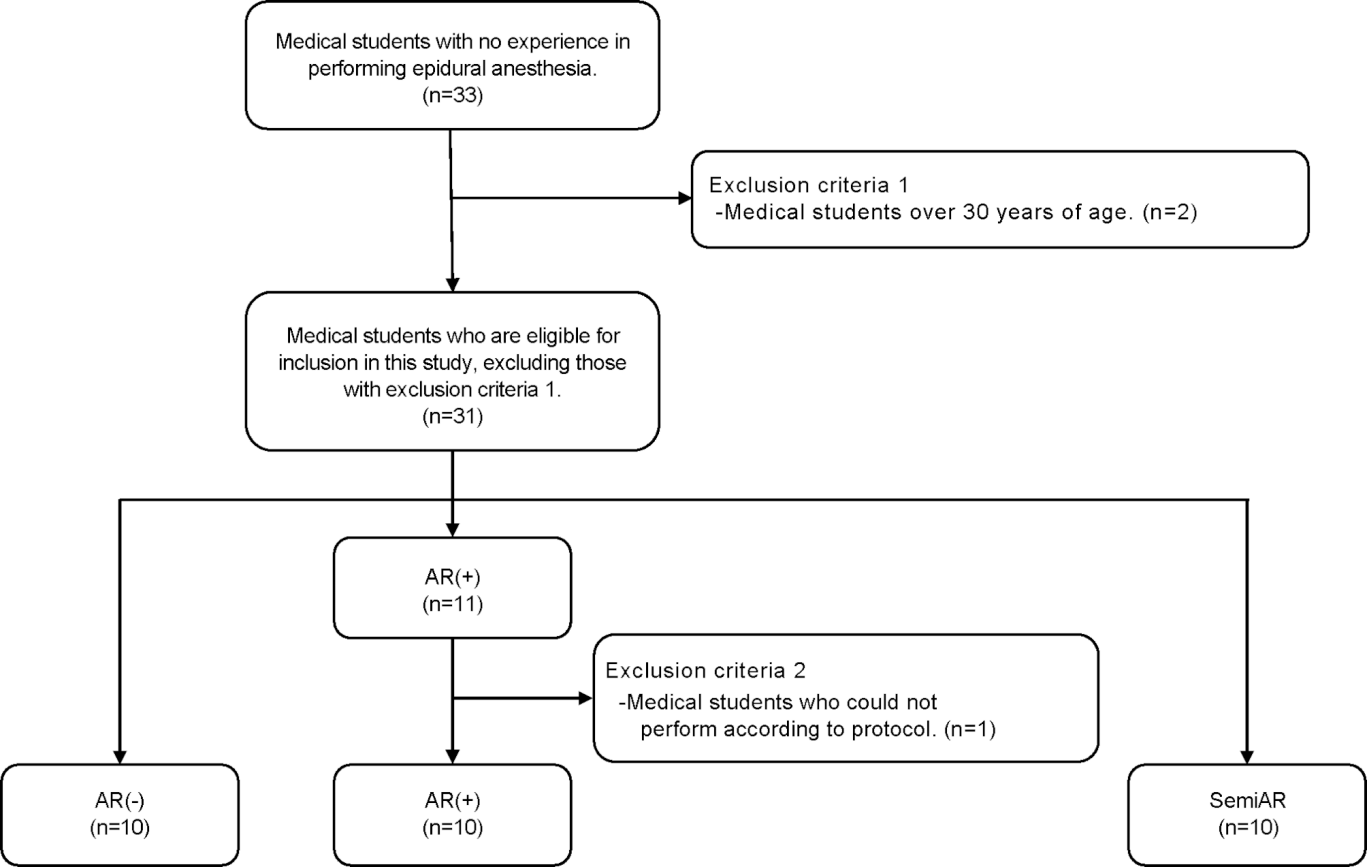
Flowchart of the target medical students. MSs (2) aged older than 30 years were excluded; MSs (1) unable to follow the protocol in the AR(+) group were excluded

**Fig. 3:**
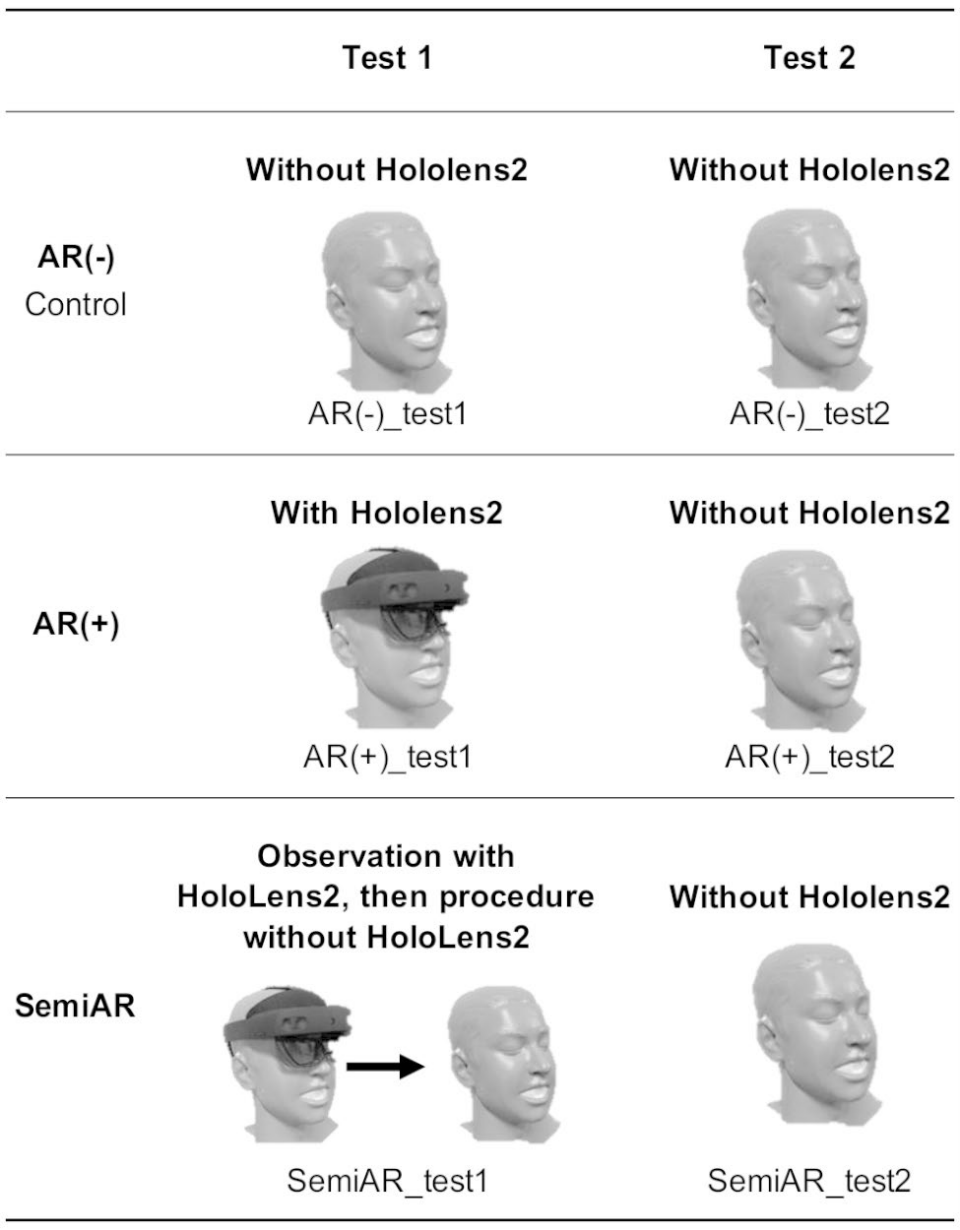
Grouping of Test 1 (first procedure) and Test 2 (second procedure). The AR(-) group performed epidural anesthesia without HoloLens2Ⓡ in both the first and second procedures. The AR(+) group performed epidural anesthesia with HoloLens2Ⓡ in the first procedure and without HoloLens2Ⓡ in the second procedure. The SemiAR group used the HoloLens2Ⓡ for 30 s to view a hologram of the ideal insertion model projected on the back of the epidural anesthesia practice kit, then removed HoloLens2Ⓡ and performed the epidural anesthesia

This study included 30 MSs with no previous experience performing epidural anesthesia undergoing bedside training at the Yamagata University Hospital between February and June 2022. In this study, MSs over the age of 30 years were excluded from the study because of the possibility that they might have had previous experience in other medical work and to avoid allowing this experience to influence the results of the study. MSs who were unable to perform the procedure according to the video lectures and procedures described below were also excluded. MSs were randomly divided into AR(-), AR(+), and semi-augmented reality (SemiAR) groups, with 10 MSs in each group (Fig. 2). In this study, the sample size was set at 10 persons per group based on previous similar studies [24]. All MSs watched a 10-min video lecture on epidural anesthesia using the paramedian approach; the AR(+) and SemiAR groups were given an additional 1-min lecture on AR-specific visualization. Figure 3 shows the grouping of Test 1 (first procedure) and Test 2 (second procedure).

### Test 1

For the first epidural puncture, epidural anesthesia was performed using the paramedian approach with the epidural anesthesia practice kit according to the following method for each group. The epidural anesthesia procedure was written on paper and placed in a visible position for the operator during the procedure. The procedure manual was designed according to Miller’s Anesthesiology [26]. The procedure time was measured from the start of the epidural needle puncture until the end of the procedure.

- The AR(-) group performed epidural anesthesia according to the procedure manual and was designated AR(-)_test1.
- In the AR(+) group, participants used HoloLens2Ⓡ after confirming its activation and observed a hologram of the ideal insertion model for 30 s. Epidural anesthesia was performed according to the procedure manual while using HoloLens2Ⓡ. This group was designated AR(+)_test1.
- In the SemiAR group, after confirming its activation, HoloLens2Ⓡ was worn, and a hologram of the ideal insertion model was observed for 30 s. HoloLens2Ⓡ was then removed, and epidural anesthesia was performed according to the procedure manual without using HoloLens2Ⓡ. This group was designated as SemiAR_test1.

Epidural anesthesia was performed using the loss of resistance method [26]. The procedure ended when the MS declared that they had finished. The needle was then cut with nippers and retained in place; the epidural puncture pad was removed from the epidural practice kit so that it was not visible to the operator. The removed epidural puncture pad was analyzed by CT to identify the surface puncture point of the spine, epidural needle angle, and epidural space puncture point. Using Blender 2.83.9 software (Blender Foundation, Amsterdam, Netherlands), the distance to the spinal puncture point between the needle of the model that the MS punctured and ideal insertion model (surface puncture point distance [SPPD]), angle difference of the needle (puncture angle [PA]), and distance to the epidural space puncture point (epidural space puncture point distance [ESPPD]) were measured. The PA was measured using the inner product of vectors (Supplementary Fig. 3). Measurements were taken by an individual who was not involved in the study and blinded to the model groups.

### Test 2

For the second epidural puncture, all groups (AR(-), AR(+), and SemiAR) performed epidural anesthesia via the paramedian approach without HoloLens2Ⓡ using the epidural anesthesia practice kit. Written instructions were provided and placed in a position visible to the operator during the procedure.

- The AR(-) group performed the epidural anesthesia again after being instructed that the puncture should be more precise than the previous one. This group was designated AR(-)_test2.
- The AR(+) group retained the image of the hologram of the ideal insertion model at Test 1, and epidural anesthesia was performed without HoloLens2Ⓡ. This group was designated AR(+)_test2.
- The SemiAR group retained the image of the hologram of the ideal insertion model at Test 1, and epidural anesthesia was performed without HoloLens2Ⓡ. This group was designated SemiAR_test2.

Data acquisition and measurements were performed as in Test 1.

### User experience questionnaire

The user experience questionnaire (UEQ) was administered to all MSs (Supplementary Table 1) [27]. MSs in the AR(-) group had no experience with HoloLens2Ⓡ; therefore, they used HoloLens2Ⓡ and observed the holograms after Test 2 was completed before completing the questionnaire. The UEQ comprised 26 questions that scored attractiveness, perspicuity, novelty, stimulation, efficiency, and dependability. The questions were scored from 1 to 7, with negative answers receiving a -3 score and positive answers a + 3 score.

### Evaluation

Figure 1E shows the surface puncture point, PA, and epidural space puncture point in the ideal insertion model. Test 1 measured the SPPD, PA, and ESPPD for the needle of the model with the MS puncture and needle of the ideal insertion model, with the AR(-) group as the control and the AR(+) and SemiAR groups as comparisons. Test 2 examined the effect of learning using AR/MR by comparing the ESPPD among the groups in Tests 1 and 2.

Dural punctures were excluded from evaluation in the epidural practice kit as anatomically improbable situations can arise (Supplementary Fig. 4). To evaluate models in which the needle could not be implanted in the epidural space, the needle was extended using Blender 2.83.9 software, and the virtual entry point into the epidural space was evaluated as the epidural space puncture point. The primary outcome was the ESPPD of Test 1 in each group, and the secondary outcomes were the SPPD of Test 1, PA of Test 1, ESPPD of Test 2, PA of Test 2, SPPD of Test 2, time required for the procedure, and UEQ in each group.

### Statistical analyses

EZR, version 1.41, was used for statistical analyses. For continuous variables, histogram visualization and the Shapiro–Wilk test were used to determine the pattern of variable distribution. The F-test was used to confirm equal variances. Continuous variables with normal distributions and equal variances were expressed as means ± standard deviations and analyzed with the Student’s t-test. Continuous variables that were not normally distributed or equally distributed were expressed as medians (interquartile range, 25–75%) and analyzed with the Mann–Whitney U test. As we believe that administering epidural anesthesia with visualization is superior to administering it blindly, a one-tailed test was used to examine the SPPD, PA, and ESPPD of the model with MS insertion and model with the needle inserted according to the textbook. One-tailed tests were also performed for the comparison of the epidural anesthesia procedure time and UEQ. Tests 1 and 2 were compared using the one-tailed Wilcoxon signed-rank sum test. P-values < 0.05 were considered statistically significant.

## Results

The mean age of the MSs was 24.0 (23.0–25.0) years (19 male; 11 female) (Supplementary Table 2); none had experience with mixed reality head-mounted smart glasses (e.g., HoloLens2Ⓡ). There were no missing values with respect to the data used to assess the participant’s needle insertion position.

### Test 1

Four MSs in AR(-)_test1, zero in AR(+)_test1, and one in SemiAR_test1 were unable to puncture the epidural space. Table 1 shows the SPPD, PA, and ESPPD for AR(-)_test1 and AR(+)_test1. Table 2 shows the SPPD, PA, and ESPPD for AR(-)_test1 and SemiAR_test1. The SPPD in AR(-)_test1 and AR(+)_test1 was 11.2 ± 4.8 and 9.1 ± 5.1 mm, respectively (P = 0.178); the PA in AR(-)_test1 and AR(+)_test1 was 15.5 ± 3.6 and 8.5 ± 4.4 degree, respectively (P < 0.01); and the ESPPD in AR(-)_test1 and AR(+)_test1 was 8.7 (5.7–14.3) and 3.5 (1.8–8.0) mm (P = 0.017), respectively. This indicates a significant difference in the PA and ESPPD (Table 1, Supplementary Fig. 5).

**Table 1:** Comparison of AR(-) and AR(+) for SPPD, PA, ESPPD, and Time in Test 1

	AR(−)_test1	AR(+)_test1	P value
SPPD [mm]	11.2 ± 4.8	9.1 ± 5.1	0.178
PA [deg]	15.5 ± 3.6	8.5 ± 4.4	< 0.01*
ESPPD [mm]	8.7 (5.7–14.3)	3.5 (1.8–8.0)	0.017*
Time [s]	117.5 (112.0–143.5)	148.0 (139.0–164.8)	0.986

Note: * P < 0.05

SPPD: Surface puncture point distance, PA: Puncture angle, ESPPD: Epidural space puncture point distance

**Table 2:** Comparison of AR(-) and SemiAR for the SPPD, PA, ESPPD, and time in Test 1

	AR(−)_test1	SemiAR_test1	P value
SPPD [mm]	11.2 ± 4.8	7.5 ± 4.2	0.04*
PA [deg]	15.5 ± 3.6	8.0 ± 4.4	< 0.01*
ESPPD [mm]	8.7 (5.7–14.3)	4.9 (3.2–5.9)	0.027*
Time [s]	117.5 (112.0–143.5)	122.0 (95.5–136.3)	0.658

Note: * P < 0.05

SPPD: Surface puncture point distance, PA: Puncture angle, ESPPD: Epidural space puncture point distance

In AR(-)_test1 and SemiAR_test1, the SPPD was 11.2 ± 4.8 and 7.5 ± 4.2 mm, respectively (P = 0.04); the PA was 15.5 ± 3.6 and 8.0 ± 4.4 degrees, respectively (P < 0.01); and the ESPPD in AR(-)_test1 and SemiAR_test1 was 8.7 (5.7–14.3) and 4.9 (3.2–5.9) mm (P = 0.027), respectively. Significant differences were observed for SPPD, PA, and ESPPD (Table 2, Supplementary Fig. 5).

**Fig. 4:**
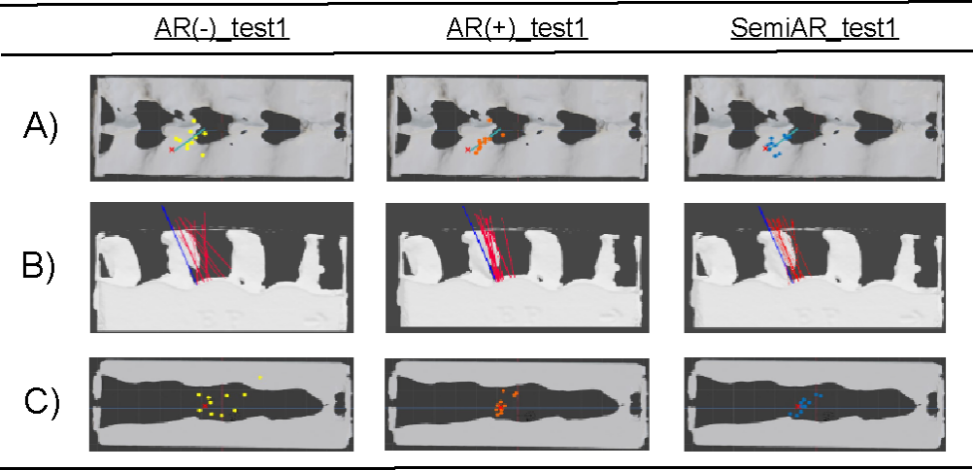
Distribution of puncture and puncture angles in Test 1. (**a**) Surface puncture point distribution for each group in Test 1. The dots indicate the puncture points punctured by the MSs. The X marks indicate the ideal surface puncture points. The blue line indicates the ideal epidural needle. (**b**) Puncture angles for each group in Test 1. The blue line indicates the ideal epidural needle puncture angle. The red line indicates the penetration angle of the epidural needle inserted by MSs. AR(-)_test1 has a variation in puncture angle, while AR(+)_test1 and SemiAR_test1 have a uniform angle close to that of the ideal needle model. (**c**) Distribution of puncture points in the epidural space for each group in Test 1. The dots indicate the epidural space puncture points punctured by MSs. The X marks indicate the ideal epidural puncture points

Figure 4 A–B shows the distribution of all surface puncture points and PAs in Test 1, respectively. AR(-)_test1 showed dispersed PAs, while AR(+)_test1 and SemiAR_test1 showed uniform PAs. Figure 4 C shows the distribution of epidural space puncture points: AR(-)_test1 was distributed, while AR(+)_test1 and SemiAR_test1 were concentrated.

The execution time of Test 1 was 117.5 (112.0–143.5) s, 148.0 (139.0–164.8) s, and 122.0 (95.5–136.3) s for the AR(-)_test1, AR(+)_test1, and SemiAR_test1, respectively. (Tables 1 and 2).

### Test 2

Test 2 evaluated whether MSs could insert the epidural needle in the appropriate position without AR/MR, as compared with Test 1. One MS in AR(-)_test2, zero in AR(+)_test2, and zero in SemiAR_test2 could not puncture the epidural space. When comparing Tests 1 and 2, there was a tendency for the ESPPD to approach the ideal insertion model in the AR(-) and SemiAR groups. However, the difference was not statistically significant (Supplementary Table 3). The ESPPD did not differ significantly between AR(-)_test2 and AR(+)_test2 (5.9 [4.0–10.6 vs. 5.0 [2.5–5.9] mm [P = 0.192]) (Table 3). However, there was a significant difference in the ESPPD between AR(-)_test2 and SemiAR_test2, 5.9 (4.0–10.6) vs. 3.4 (2.5–5.5) mm (P = 0.045) (Table 4).

**Table 3:** Comparison of AR(-) and AR(+) for the SPPD, PA, ESPPD, and Time in Test 2

	AR(-)_test2	AR(+)_test2	P value
SPPD [mm]	9.1 (5.6–17.2)	10.5 (5.0–13.5)	0.325
PA [deg]	9.7 (6.0–17.1)	9.9 (6.6–10.7)	0.315
ESPPD [mm]	5.9 (4.0–10.6)	5.0 (2.5–5.9)	0.192
Time [s]	100.0 (79.5–128.5)	115.0 (97.3–143.0)	0.88

Note: * P < 0.05

SPPD: Surface puncture point distance, PA: Puncture angle, ESPPD: Epidural space puncture point distance

**Table 4:** Comparison of AR(-) and SemiAR for the SPPD, PA, ESPPD, and Time in Test 2

	AR(-)_test2	SemiAR_test2	P value
SPPD [mm]	9.1 (5.6–17.2)	7.1 (4.9–10.3)	0.157
PA [deg]	9.7 (6.0–17.1)	7.3 (6.4–9.7)	0.176
ESPPD [mm]	5.9 (4.0–10.6)	3.4 (2.5–5.5)	0.045*
Time [s]	100.0 (79.5–128.5)	93.0 (79.8–159.5)	0.381

Note: * P < 0.05

SPPD: Surface puncture point distance, PA: Puncture angle, ESPPD: Epidural space puncture point distance

The execution time for Test 2 was 100.0 (79.5–128.5) s, 115.0 (97.3–143.0) s, and 93.0 (79.8-159.5) s for AR(-)_test2, AR(+)_test2, and SemiAR_test2, respectively (Tables 3 and 4). The total time for Tests 1 and 2 was 226.5 (201.0-251.5) s for the AR(-) group, 288.5 (239.5-382.3) s for the AR(+) group, and 232.5 (211.0-293.0) s for the SemiAR group (Tables 5 and 6).

**Table 5:** Comparison of AR(-) and AR(+) for time in Test 1 and time in Test 2, as well as total time in Tests 1 and 2

	AR(-)	AR(+)	P value
Time in Test 1	117.5 (112.0–143.5)	148.0 (139.0–164.8)	0.986
Time in Test 2	100.0 (79.5–128.5)	115.0 (97.3–143.0)	0.88
Total time in Tests 1 and 2	226.5 (201.0–251.5)	288.5 (239.5–382.3)	0.992

**Table 6:** Comparison of AR(-) and SemiAR for time in Test 1 and time in Test 2, as well as total time in Tests 1 and 2

	AR(-)	SemiAR	P value
Time in Test 1	117.5 (112.0–143.5)	122.0 (95.5–136.3)	0.658
Time in Test 2	100.0 (79.5–128.5)	93.0 (79.8–159.5)	0.381
Total time in Tests 1 and 2	226.5 (201.0–251.5)	232.5 (211.0–293.0)	0.688

### Results of the user experience questionnaire

The AR(+) group had significantly more positive answers to questions in the perspicuity, novelty, stimulation, dependability, and efficiency categories of the UEQ than the AR(-) group. The SemiAR group also provided significantly more positive responses in all items of the UEQ than the AR(-) group (Supplementary Fig. 6).

## Discussion

In Test 1, AR(+)_test1 MSs were able to place the epidural needle close to the ideal locations for the PA and ESPPD. In SemiAR_test1, the epidural needle was placed close to the ideal needle in all cases (SPPD, PA, and ESPPD).

These results indicate that epidural anesthesia with AR may be more appropriate for epidural needle placement than epidural anesthesia with textbook or video learning alone. The large variance in the SPPD for the AR(+)_test1 MSs may be attributed to none of the participants having experience using mixed reality head-mounted smart glasses; thus, the MSs may have misidentified the surface puncture point because they had not mastered AR sensation. Alternatively, the registration accuracy, and inherent overlay accuracy and stability could have also had some impact on the variance in the SPPD [28].

Test 2 resulted in the SemiAR_test2 group placing the epidural needle closer to the ideal needle model at the epidural space puncture point compared to AR(-)_test2. The SemiAR group performed image training using a HoloLens2Ⓡ and performed epidural anesthesia without AR in both Tests 1 and 2. This tended to improve epidural anesthesia technique.

However, a comparison of AR(+)_test1 and AR(+)_test2 showed no trend toward successful epidural anesthesia. This could be attributed to the fact that the AR(+) group used HoloLens2Ⓡ in Test 1 and did not use it in Test 2, resulting in a significantly different environment between the first and second sessions.

The UEQ results indicate that many students had a positive perception of AR. We believe that AR is a useful learning tool in terms of attracting students’ interest. In terms of time, the results of Tests 1 and 2 showed that the use of AR/MR technology did not contribute to a reduction in procedure time.

A previous study has examined the possibility of epidural anesthesia using AR [29]. However, ours is the first to compare the accuracy of epidural anesthesia performed by MSs with no epidural anesthesia experience using AR with that of conventional epidural anesthesia. This study suggests that AR/MR has the potential to aid in the understanding of anatomical structures and improve epidural anesthesia techniques [30].

There is no practice in clinical practice, it is always an actual procedure, which in the case of this study is the Test 1 situation. In Test 1, the SemiAR group had the best procedure accuracy, suggesting that epidural anesthesia using AR/MR technology may be effective for image training before the actual surgery. For residents inexperienced in epidural anesthesia, AR/MR technology appears to be a sufficient tool to learn the optimal insertion angle technique when “walking” the needle tip. Since most patients undergoing thoracic and abdominal surgery undergo CT, we believe that AR/MR technology can be applied clinically and that previewing epidural approaches with actual patient CT images before performing epidural anesthesia promotes safety. Furthermore, epidural anesthesia AR/MR is expected to be used by experienced anesthesiologists as a technical learning tool to perform epidural anesthesia more accurately and safely.

Some HoloLens2Ⓡ medical applications have been approved by the US Food and Drug Administration. In the future, epidural anesthesia may be performed with HoloLens2Ⓡ, thereby improving the understanding of the anatomy and safety of epidural anesthesia. This will facilitate epidural anesthesia in patients with obesity and spinal stenosis, for whom locating the epidural space is difficult, and avoid unintentional dural and vascular puncture by the epidural needle.

### Limitations

There are eight limitations of this study. First, the commercially available phantoms are practice kits for epidural anesthesia and have a larger gap than the actual vertebral arch, which can cause the epidural needle to accidentally penetrate the epidural space puncture point. Future research should be conducted using epidural anesthesia practice kits with smaller gaps or epidural anesthesia AR/MR in actual clinical practice. Second, blinding was impossible for MSs. Third, the sample size was determined with reference to previous similar studies. Increasing the sample size in future studies may provide more reliable results. Fourth, the AR markers were used to visualize the virtual spine, and the epidural anesthesia practice kit was held in place to prevent misalignment; however, the pressure applied by the participant during the puncture may have caused some movement, resulting in misalignment of the virtual spine. Fifth, we did not consider the possibility that the needle could successfully approach the epidural space even when the surface puncture point was located far from the ideal surface puncture point in this study. In clinical practice, the needle can approach the epidural space by changing the angle of the needle, even when the insertion point is located far from the ideal point. This study determined how close the epidural needle can be inserted to the model entry point described in Miller’s Anesthesiology (textbook), which is currently considered ideal. Therefore, there may be differences in clinical situations. We believe it is important to continue our research to further improve the practicality of applying AR/MR technology while validating it in clinical situations. Sixth, it may be necessary to consider the time factor in the data acquisition process. In the AR(-) group, there was a period of time between the video lecture and start of epidural anesthesia. In the AR(+) and SemiAR groups, there was a period of time between the video lecture and start of hologram observation, and a period of time between the end of hologram observation and start of epidural anesthesia. Thus, there were different gaps in time periods in each group. In addition, although we took care to avoid time gaps in timing, such as the replacement of the epidural puncture pad in all groups, there could have been a difference of a few seconds in each group. Seventh is an examination of the time factor when AR/MR technology is applied in actual clinical practice. Although AR/MR data acquisition, processing, and registration are likely to be required in actual clinical settings, these times were not measured in this study. We have confirmed that it takes approximately 1 min from the time the HoloLens2Ⓡ is turned on to the time the application is launched, although it is necessary to examine the exact time required in actual clinical settings. Eighth, we have not examined the comparison between the administration of epidural anesthesia using AR/MR technology and other technologies. In future studies, it would be appropriate to consider comparisons with other techniques such as Fiducial Registered CT imaging and ultrasound-guided access in future studies.

## Conclusions

These findings suggest that AR/MR technology is a useful tool to construct an image of epidural anesthesia and can significantly contribute to improved epidural anesthesia skills, potentially stabilizing patient conditions, and solving medically important epidural anesthesia problems.

## Electronic supplementary material

Below is the link to the electronic supplementary material.

Supplementary Table 1: Contents of the user experience questionnaire (UEQ)

Supplementary Table 2: Characteristics of each group of medical students with no experience with epidural anesthesia

Supplementary Table 3: Comparison of the ESPPD between Tests 1 and 2 in all groups

Supplementary Figure 1: CT scanner image of the ideal insertion model:  CT scanner images of the ideal insertion model from the side, top, and front views

Supplementary Figure 2: Posture of epidural anesthesia operator (without and with HoloLens2Ⓡ) : The epidural anesthesia operator wearing HoloLens2Ⓡ can observe the hologram of the ideal insertion model projected on the back of the epidural anesthesia practice kit

Supplementary Figure 3: Formula for the inner product of vectors:  The epidural needle puncture angle of the ideal insertion model and the participants’ epidural needle puncture angle were calculated using the inner product of vectors

Supplementary Figure 4: Evaluation of dural puncture and internal structure of epidural anesthesia practice kit: Dural punctures were excluded from the evaluation of the epidural anesthesia practice kit because of the possibility of anatomically improbable puncture situations, as indicated by the arrows

Supplementary Figure 5: Comparison of the SPPD, PA, and ESPPD in AR(+)_test1 and SemiAR_test1, with AR(-)_test1 as the control group:  Regarding the SPPD, SemiAR_test1 had a surface puncture point closer to the ideal needle model (A2). Regarding the PA, both AR(+)_test1 and SemiAR_test1 had a puncture angle close to the ideal needle model (B1, B2). Regarding the ESPPD, both the AR(+)_test1 and SemiAR_test1 groups had epidural space puncture points closer to the ideal needle model (C1, C2). Note: * indicates significant between-group difference (P<0.05)

Supplementary Figure 6: UEQ results for the AR(-) group as the control group:  The AR(+) group had significantly more positive answers to questions in the perspicuity, novelty, stimulation, dependability, and efficiency categories of the UEQ than the AR(-) group. The SemiAR group also obtained significantly more positive responses in all items of the UEQ than the AR(-) group. Note: * indicates a significant between-group difference (P<0.05)

## Data Availability

The data underlying this article will be shared on reasonable request to the corresponding author.

## Abbreviations

AR/MR: Augmented reality/mixed reality
CT: Computed tomography
ESPPD: Epidural space puncture point distance
MSs: Medical students
PA: Puncture angle
SemiAR: Semi-augmented reality
SPPD: Surface puncture point distance
UEQ: User experience questionnaire
