# Supplementary material for: Comparison of accuracy between augmented reality/mixed reality techniques and conventional techniques for epidural anesthesia using a practice phantom model kit
Source: BMC Anesthesiol. 2023 May 20;23:171. doi: 10.1186/s12871-023-02133-w (PMC10199582; doi:10.1186/s12871-023-02133-w)
Supplement: Supplementary file 1 — Supplementary Table 1: Contents of the user experience questionnaire (UEQ) [file 12871_2023_2133_MOESM1_ESM.doc]

Supplementary Table 1. Contents of the user experience questionnaire (UEQ)

|  | **1** | **2** | **3** | **4** | **5** | **6** | **7** |  |  |
| --- | --- | --- | --- | --- | --- | --- | --- | --- | --- |
| Annoying |  |  |  |  |  |  |  | Enjoyable | Attractiveness |
| Not understandable |  |  |  |  |  |  |  | Understandable | Perspicuity |
| Creative |  |  |  |  |  |  |  | Dull | Novelty |
| Easy to learn |  |  |  |  |  |  |  | Difficult to learn | Perspicuity |
| Valuable |  |  |  |  |  |  |  | Inferior | Stimulation |
| Boring |  |  |  |  |  |  |  | Exciting | Stimulation |
| Not interesting |  |  |  |  |  |  |  | Interesting | Stimulation |
| Unpredictable |  |  |  |  |  |  |  | Predictable | Dependability |
| Fast |  |  |  |  |  |  |  | Slow | Efficiency |
| Inventive |  |  |  |  |  |  |  | Conventional | Novelty |
| Obstructive |  |  |  |  |  |  |  | Supportive | Dependability |
| Good |  |  |  |  |  |  |  | Bad | Attractiveness |
| Complicated |  |  |  |  |  |  |  | Easy | Perspicuity |
| Unlikable |  |  |  |  |  |  |  | Pleasing | Attractiveness |
| Usual |  |  |  |  |  |  |  | Leading edge | Novelty |
| Unpleasant |  |  |  |  |  |  |  | Pleasant | Attractiveness |
| Secure |  |  |  |  |  |  |  | Not secure | Dependability |
| Motivating |  |  |  |  |  |  |  | Demotivating | Stimulation |
| Meets expectations |  |  |  |  |  |  |  | Does not meet expectations | Dependability |
| Inefficient |  |  |  |  |  |  |  | Efficient | Efficiency |
| Clear |  |  |  |  |  |  |  | Confusing | Perspicuity |
| Impractical |  |  |  |  |  |  |  | Practical | Efficiency |
| Organized |  |  |  |  |  |  |  | Cluttered | Efficiency |
| Attractive |  |  |  |  |  |  |  | Unattractive | Attractiveness |
| Friendly |  |  |  |  |  |  |  | unfriendly | Attractiveness |
| Conservative |  |  |  |  |  |  |  | Innovative | Novelty |

The UEQ comprises 26 questions and scores attractiveness, clarity, novelty, stimulation, efficiency, and reliability. Participants answered the 26 questions on a scale of 1 to 7. Each question fits one of the five properties and is scored -3 points for a negative answer and +3 points for a positive answer. An average score was calculated for each property.
