# Supplementary material for: Comparison of accuracy between augmented reality/mixed reality techniques and conventional techniques for epidural anesthesia using a practice phantom model kit
Source: BMC Anesthesiol. 2023 May 20;23:171. doi: 10.1186/s12871-023-02133-w (PMC10199582; doi:10.1186/s12871-023-02133-w)
Supplement: Supplementary file 2 — Supplementary Table 2: Characteristics of each group of medical students with no experience with epidural anesthesia [file 12871_2023_2133_MOESM2_ESM.doc]

Supplementary Table 2. Characteristics of each group of medical students with no experience with epidural anesthesia

|  | Age | Male/Female |
| --- | --- | --- |
| AR(－) | 24.5 (22.5–25.8) | 6/4 |
| AR(＋) | 23.0 (23.0–24.5) | 6/4 |
| SemiAR | 24.0 (23.0–26.3) | 7/3 |
| Total | 24.0 (23.0–25.0) | 19/11 |

The mean age of the medical students was 24.0 (23.0–25.0) years. There were 19 male and 11 female individuals.
