# Supplementary material for: Comparison of accuracy between augmented reality/mixed reality techniques and conventional techniques for epidural anesthesia using a practice phantom model kit
Source: BMC Anesthesiol. 2023 May 20;23:171. doi: 10.1186/s12871-023-02133-w (PMC10199582; doi:10.1186/s12871-023-02133-w)
Supplement: Supplementary file 3 — Supplementary Table 3: Comparison of the ESPPD between Tests 1 and 2 in all groups [file 12871_2023_2133_MOESM3_ESM.doc]

Supplementary Table 3. Comparison of the ESPPD between Tests 1 and 2 in all groups

|  | Test 1 | Test 2 | P value |
| --- | --- | --- | --- |
| AR(－) | 8.7 (5.7–14.3) | 5.9 (4.0–10.6) | 0.097 |
| AR(＋) | 3.5 (1.8–8.0) | 5.0 (2.5–5.9) | 0.807 |
| SemiAR | 4.9 (3.2–5.9) | 3.4 (2.5–5.5) | 0.053 |

When comparing Tests 1 and 2 of the ESPPD for all groups, there was a tendency for the ESPPD to approach the ideal insertion model in the AR(-) and SemiAR groups, although the difference was not significant.
