# Supplementary material for: Comparison of accuracy between augmented reality/mixed reality techniques and conventional techniques for epidural anesthesia using a practice phantom model kit
Source: BMC Anesthesiol. 2023 May 20;23:171. doi: 10.1186/s12871-023-02133-w (PMC10199582; doi:10.1186/s12871-023-02133-w)
Supplement: Supplementary file 4 — Supplementary Figure 1: CT scanner image of the ideal insertion model: CT scanner images of the ideal insertion model from the side, top, and front views [file 12871_2023_2133_MOESM4_ESM.doc]

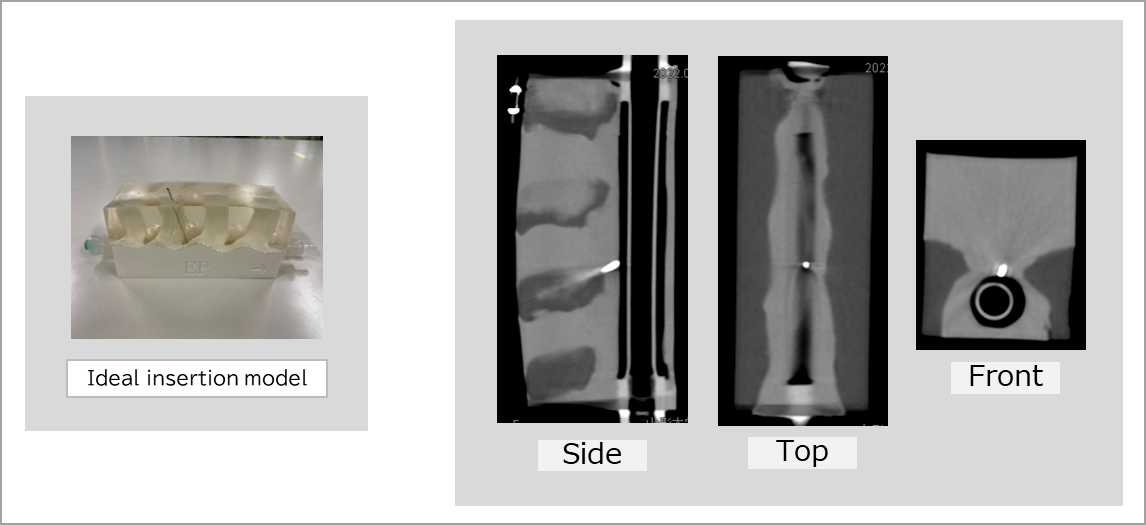


Supplementary Figure 1: CT scanner image of the ideal insertion model

CT scanner images of the ideal insertion model from the side, top, and front views.
