# Supplementary material for: Comparison of accuracy between augmented reality/mixed reality techniques and conventional techniques for epidural anesthesia using a practice phantom model kit
Source: BMC Anesthesiol. 2023 May 20;23:171. doi: 10.1186/s12871-023-02133-w (PMC10199582; doi:10.1186/s12871-023-02133-w)
Supplement: Supplementary file 5 — Supplementary Figure 2: Posture of epidural anesthesia operator (without and with HoloLens2Ⓡ) : The epidural anesthesia operator wearing HoloLens2Ⓡ can observe the hologram of the ideal insertion model projected on the back of the epidural anesthesia practice kit [file 12871_2023_2133_MOESM5_ESM.doc]

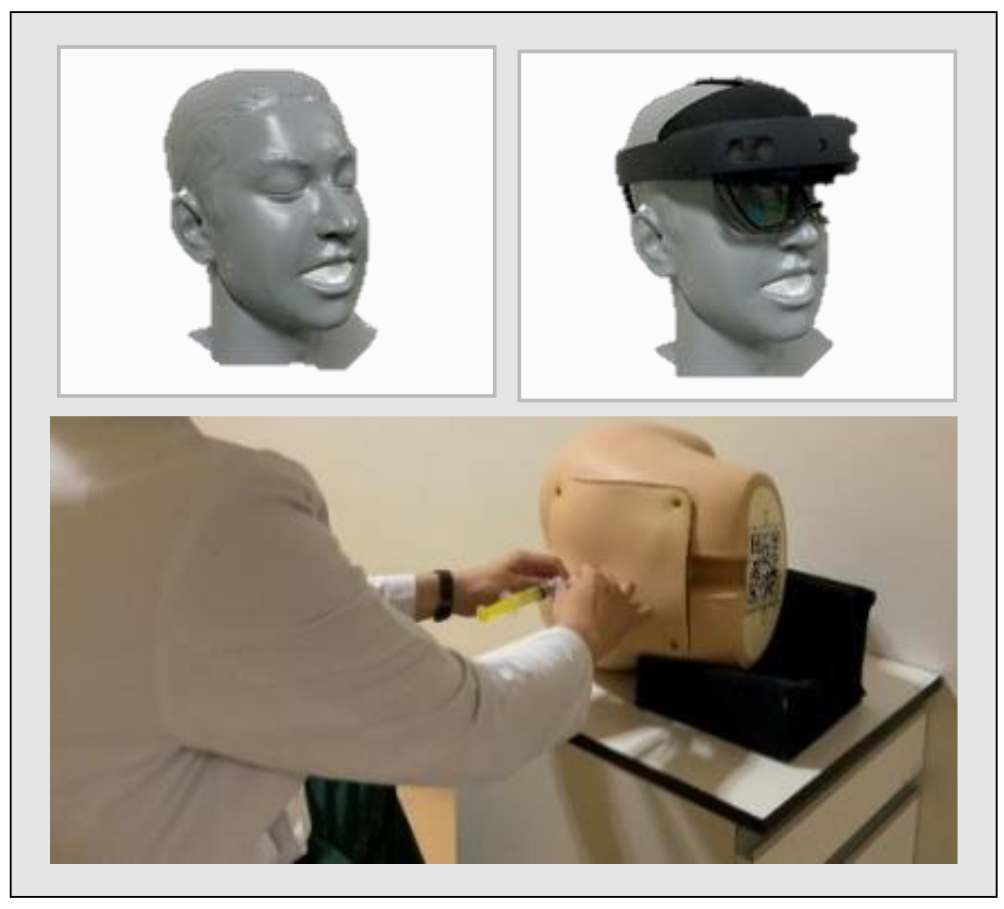


Supplementary Figure 2: Posture of epidural anesthesia operator (without and with HoloLens2®)

The epidural anesthesia operator wearing HoloLens2® can observe the hologram of the ideal insertion model projected on the back of the epidural anesthesia practice kit.
