# Supplementary material for: Comparison of accuracy between augmented reality/mixed reality techniques and conventional techniques for epidural anesthesia using a practice phantom model kit
Source: BMC Anesthesiol. 2023 May 20;23:171. doi: 10.1186/s12871-023-02133-w (PMC10199582; doi:10.1186/s12871-023-02133-w)
Supplement: Supplementary file 6 — Supplementary Figure 3: Formula for the inner product of vectors: The epidural needle puncture angle of the ideal insertion model and the participants’ epidural needle puncture angle were calculated using the inner product of vectors [file 12871_2023_2133_MOESM6_ESM.doc]

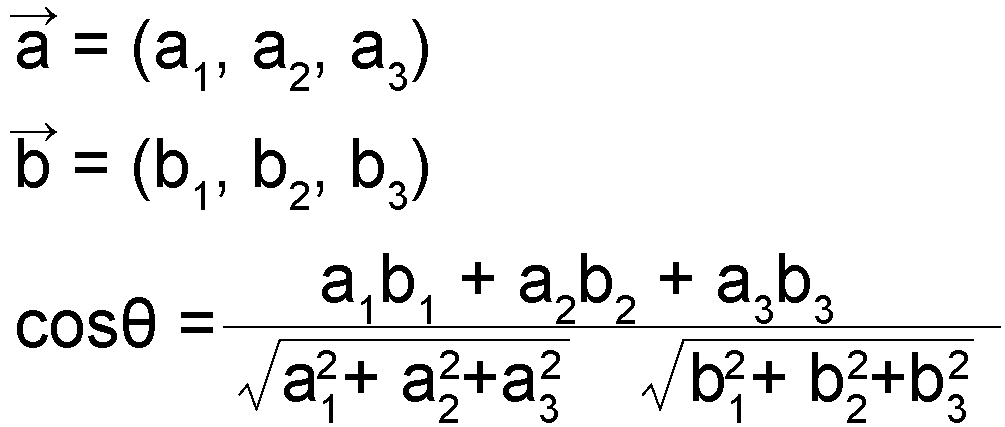


Supplementary Figure 3: Formula for the inner product of vectors

The epidural needle puncture angle of the ideal insertion model and the participants’ epidural needle puncture angle were calculated using the inner product of vectors.
