# Supplementary material for: Comparison of accuracy between augmented reality/mixed reality techniques and conventional techniques for epidural anesthesia using a practice phantom model kit
Source: BMC Anesthesiol. 2023 May 20;23:171. doi: 10.1186/s12871-023-02133-w (PMC10199582; doi:10.1186/s12871-023-02133-w)
Supplement: Supplementary file 7 — Supplementary Figure 4: Evaluation of dural puncture and internal structure of epidural anesthesia practice kit: Dural punctures were excluded from the evaluation of the epidural anesthesia practice kit because of the possibility of anatomically improbable puncture situations, as indicated by the arrows [file 12871_2023_2133_MOESM7_ESM.doc]

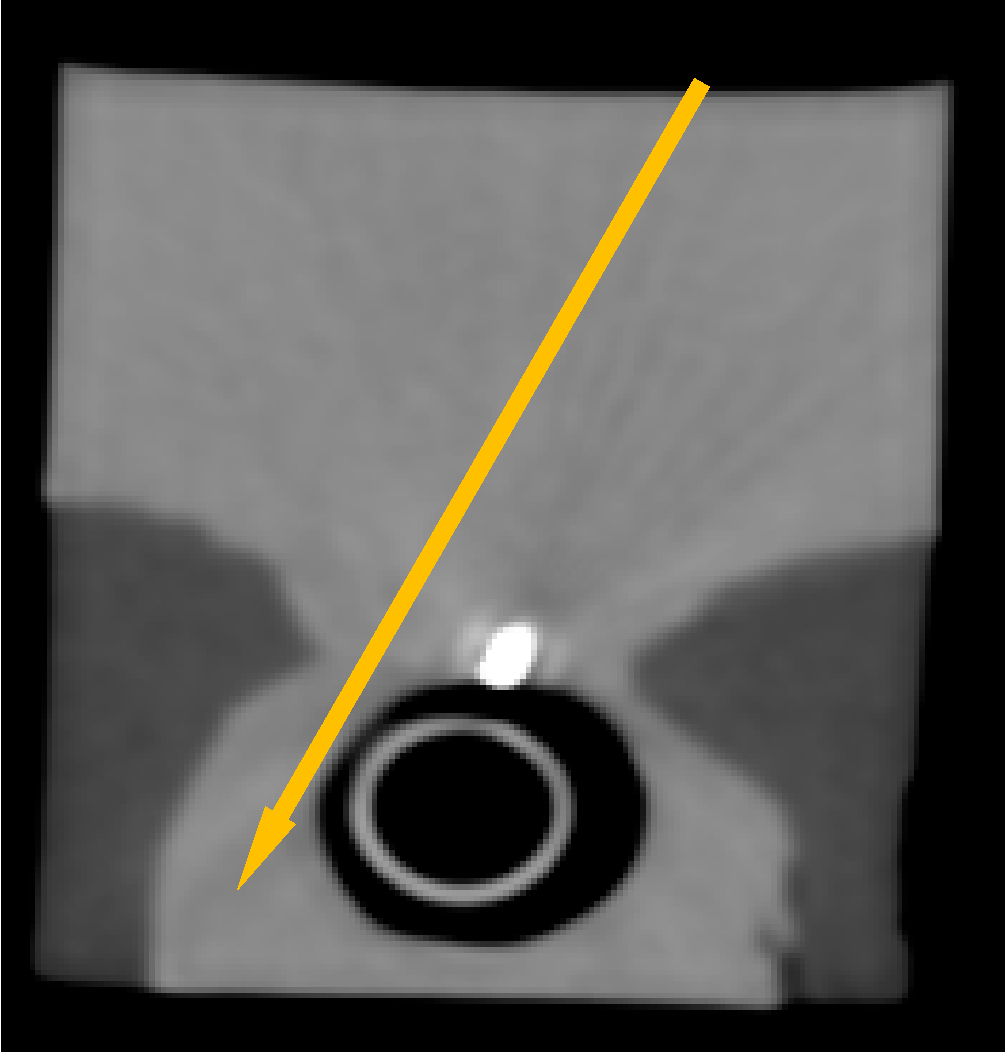


Supplementary Figure 4: Evaluation of dural puncture and internal structure of epidural anesthesia practice kit

Dural punctures were excluded from the evaluation of the epidural anesthesia practice kit because of the possibility of anatomically improbable puncture situations, as indicated by the arrows.
