# Supplementary material for: Comparison of accuracy between augmented reality/mixed reality techniques and conventional techniques for epidural anesthesia using a practice phantom model kit
Source: BMC Anesthesiol. 2023 May 20;23:171. doi: 10.1186/s12871-023-02133-w (PMC10199582; doi:10.1186/s12871-023-02133-w)
Supplement: Supplementary file 8 — Supplementary Figure 5: Comparison of the SPPD, PA, and ESPPD in AR(+)_test1 and SemiAR_test1, with AR(-)_test1 as the control group: Regarding the SPPD, SemiAR_test1 had a surface puncture point closer to the ideal needle model (A2). Regarding the PA, both AR(+)_test1 and SemiAR_test1 had a puncture angle close to the ideal needle model (B1, B2). Regarding the ESPPD, both the AR(+)_test1 and SemiAR_test1 groups had epidural space puncture points closer to the ideal needle model (C1, C2). Note: * indicates significant between-group difference (P<0.05) [file 12871_2023_2133_MOESM8_ESM.doc]

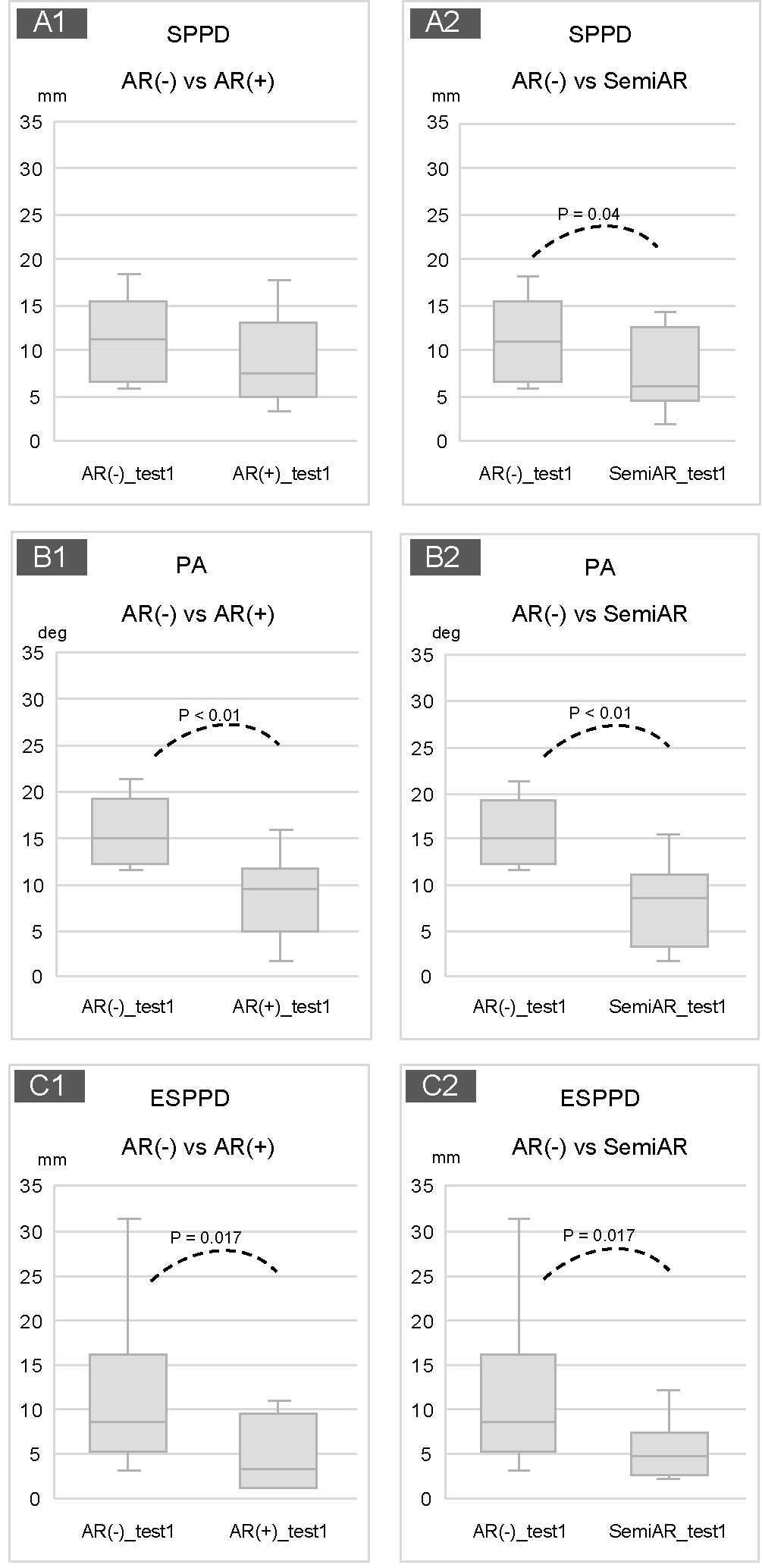


Supplementary Figure 5: Comparison of the SPPD, PA, and ESPPD in AR(+)_test1 and SemiAR_test1, with AR(-)_test1 as the control group. Regarding the SPPD, SemiAR_test1 had a surface puncture point closer to the ideal needle model (A2). Regarding the PA, both AR(+)_test1 and SemiAR_test1 had a puncture angle close to the ideal needle model (B1, B2). Regarding the ESPPD, both the AR(+)_test1 and SemiAR_test1 groups had epidural space puncture points closer to the ideal needle model (C1, C2). Note: ∗ indicates significant between-group difference (P<0.05).
