# Supplementary material for: Comparison of accuracy between augmented reality/mixed reality techniques and conventional techniques for epidural anesthesia using a practice phantom model kit
Source: BMC Anesthesiol. 2023 May 20;23:171. doi: 10.1186/s12871-023-02133-w (PMC10199582; doi:10.1186/s12871-023-02133-w)
Supplement: Supplementary file 9 — Supplementary Figure 6: UEQ results for the AR(-) group as the control group: The AR(+) group had significantly more positive answers to questions in the perspicuity, novelty, stimulation, dependability, and efficiency categories of the UEQ than the AR(-) group. The SemiAR group also obtained significantly more positive responses in all items of the UEQ than the AR(-) group. Note: * indicates a significant between-group difference (P<0.05) [file 12871_2023_2133_MOESM9_ESM.doc]

Supplementary Figure 6. UEQ results for the AR(-) group as the control group

The AR(+) group had significantly more positive answers to questions in the perspicuity, novelty, stimulation, dependability, and efficiency categories of the UEQ than the AR(-) group. The SemiAR group also obtained significantly more positive responses in all items of the UEQ than the AR(-) group. Note: ∗ indicates a significant between-group difference (P<0.05).
